# Medication Burden and Adherence of Antiretroviral Therapy Among Older People Living with HIV in the Context of Multimorbidity and Polypharmacy: A Multicenter Study

**DOI:** 10.3390/v18030387

**Published:** 2026-03-20

**Authors:** Yaqin Zhou, Hong Zuo, Sitong Luo, Chunyuan Zheng, Honghong Wang

**Affiliations:** 1 Vanke School of Public Health, Tsinghua University, Beijing 100084, China; yaqinzhou99@hotmail.com; 2 Xiangya School of Nursing, Central South University, Changsha 410013, China; zuohong001@hotmail.com

**Keywords:** HIV, aging, multimorbidity, polypharmacy, medication burden, antiretroviral therapy, adherence

## Abstract

Background: Population aging among people living with HIV (PLWH) has led to a growing burden of multimorbidity and complex medication regimens. However, the relationships between medication-related challenges and antiretroviral therapy (ART) adherence in older PLWH remain insufficiently understood. Methods: A multicenter cross-sectional study was conducted among PLWH aged ≥50 years receiving routine HIV care in Hunan Province, China. Multimorbidity, polypharmacy, potential drug–drug interactions (PDDIs), medication-related burden, and ART adherence were assessed using validated instruments and clinical records. Path analysis was applied to examine hypothesized relationships based on the transactional model of stress and coping. Results: Among 301 participants, 54.2% experienced multimorbidity and 29.2% met criteria for polypharmacy. Medication-related burden was moderate to high. The proposed path model demonstrated good fit. Multimorbidity was positively associated with polypharmacy and PDDIs, both of which contributed to higher medication-related burden. Medication-related burden was the only factor directly associated with lower ART adherence, whereas polypharmacy and PDDIs showed no significant direct effects. Conclusions: Medication-related burden was significantly associated with both clinical complexity indicators and ART adherence among older PLWH. Interventions addressing patients’ subjective treatment burden may be critical for sustaining long-term adherence in aging HIV populations.

## 1. Introduction

The widespread availability of antiretroviral therapy (ART) has transformed HIV infection into a chronic, manageable condition and substantially extended life expectancy among people living with HIV (PLWH). As a result, the global HIV population is rapidly aging [1]. It is estimated that by 2030, more than 70% of PLWH in many settings will be aged 50 years or older, with similar trends emerging in China and other Asian countries [2]. Aging with HIV is accompanied by a disproportionate burden of non-communicable diseases, driven by immune senescence, chronic inflammation, and long-term ART exposure [3].

Multimorbidity is highly prevalent among older PLWH and often necessitates long-term pharmacological treatment beyond ART [4]. Polypharmacy increases regimen complexity and elevates the risk of medication-related problems, particularly in HIV treatment, where antiretroviral agents frequently share metabolic pathways with medications prescribed for cardiovascular, metabolic, and neuropsychiatric conditions. A major clinical concern arising from polypharmacy is the occurrence of potential drug–drug interactions (PDDIs) [5]. Interactions between ART and non-ART medications may compromise therapeutic efficacy or increase toxicity. Older PLWH are especially vulnerable due to age-related changes in renal and hepatic function [6]. While the pharmacological consequences of PDDIs are well documented, less is known about how medication complexity and interaction risk translate into behavioral outcomes such as ART adherence.

Beyond objective indicators, medication-related burden represents a critical but understudied dimension of treatment complexity. Medication burden reflects patients’ subjective experiences of medication use, including concerns about side effects, daily life interference, practical difficulties, financial strain, and perceived treatment overload [7]. Evidence from other chronic disease populations suggests that high medication burden may undermine long-term adherence [8]. However, its role in HIV care, particularly among older adults, remains insufficiently explored.

The transactional model of stress and coping provides a useful theoretical framework for understanding associations between clinical complexity and patient behavior [9]. According to this model, stress arises not solely from objective demands but from individuals’ cognitive appraisal of those demands and their perceived ability to cope. Applied to HIV care, multimorbidity and polypharmacy are conceptualized as medication-related stressors that are associated with adherence behavior.

**Figure 1:**
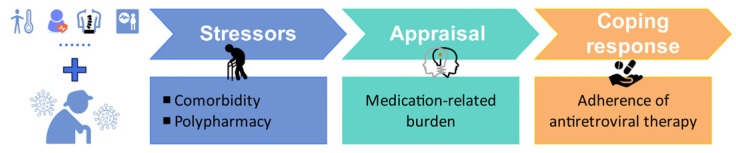
A theoretical framework of medication-related stress and coping among older people living with HIV, adapted from the transactional model of stress and coping.

Accordingly, the present study aimed to examine the relationships among multimorbidity, polypharmacy, medication burden, and ART adherence among older PLWH. Guided by the transactional model of stress and coping, we developed a conceptual framework to illustrate the hypothesized relationships among comorbidity, polypharmacy, and medication-related burden among older PLWH (Figure 1). In this study, we hypothesized that health-related conditions common in older adults, such as multimorbidity and polypharmacy, accumulate to form medication-related stressors. Medication-related burden was hypothesized to be associated with both clinical complexity and ART adherence.

## 2. Materials and Methods

### 2.1. Study Design and Sample

This multicenter cross-sectional study was conducted between September 2020 and January 2023 in Hunan Province, China. The study population consisted of PLWH aged 50 years and older who were receiving routine HIV care. Participants were recruited from two major HIV care providers in the region, the Yuelu District Center for Disease Control and the HIV outpatient clinic of Changsha First Hospital. Both institutions provide standardized ART management and long-term follow-up care services.

Participants were enrolled using a successive sampling method during routine clinic visits. Eligibility criteria included: (1) laboratory-confirmed HIV infection according to national diagnostic guidelines; (2) age ≥ 50 years; and (3) current use of ART with a stable regimen. Individuals with documented cognitive impairment or severe sensory deficits that could hinder comprehension of the questionnaire or meaningful participation were excluded. All participants provided written informed consent before enrollment. The study protocol was approved by the institutional ethics committee and conducted in accordance with ethical standards.

Sample size estimation was guided by the research purpose and the complexity of the proposed path model. For models including approximately five observed variables, a minimum sample size of 100 has been suggested; however, parameter estimates and statistical power are considered unstable when sample sizes fall below 200 [10]. To ensure adequate model stability and statistical power, and accounting for an anticipated 20% rate of invalid or incomplete questionnaires, a minimum sample size of 250 participants was required.

### 2.2. Variables and Measures

Sociodemographic characteristics, including sex, age, residence, marital status, and monthly income, were collected using a self-designed questionnaire.

Clinical characteristics, including comorbid conditions, were assessed through self-report and subsequently verified using electronic medical records. Specifically, multimorbidity was defined as the presence of two or more chronic conditions coexisting in the same individual. Medication use was comprehensively documented, encompassing all antiretroviral drugs, medications prescribed for comorbid conditions, and self-initiated products such as dietary supplements and traditional Chinese medicines. When detailed composition information was available, herbal products and traditional Chinese medicines were also evaluated for potential drug–drug interactions. Each distinct pill or formulation was counted as a separate medication. Polypharmacy was defined as the concurrent use of five or more medications, which reflects the quantitative extent of medication exposure.

PDDIs were evaluated using the Liverpool HIV Drug Interactions database (University of Liverpool, Liverpool, UK; https://www.hiv-druginteractions.org, accessed on 25 January 2023), an internationally validated and regularly updated HIV-specific interaction resource. PDDIs represent pharmacological interaction risk arising from specific drug combinations. To ensure accuracy, identified interactions were independently reviewed and confirmed by experienced HIV clinicians at each participating site. The use of a single standardized database ensured consistency in interaction grading across centers. All combinations of ART and non-ART medications were systematically screened and classified as red (contraindicated), amber (potential interaction requiring monitoring), yellow (weak interaction), or green (no interaction). For analytical purposes, PDDIs were defined as the presence of at least one red, amber, or yellow interaction.

Medication-related burden was assessed using the Chinese version of the Living with Medicines Questionnaire version 3 (C-LMQ-3) [11]. The scale consists of 39 items across eight domains and uses a five-point Likert scale. Total scores range from 39 to 195, with higher scores indicating greater perceived medication burden. Medication-related burden reflects patients’ subjective appraisal of treatment demands and complexity.

ART adherence was measured using the Center for Adherence Support Evaluation (CASE) Adherence Index, a validated three-item self-report measure [12]. The CASE index evaluates overall missed doses, average weekly missed doses, and recency of missed doses. Total scores range from 3 to 16.

### 2.3. Data Collection

Data were collected through structured, interviewer-assisted questionnaire administration to accommodate the cognitive, visual, and literacy needs of older adults. All participants completed the survey in a private consultation room to ensure confidentiality and minimize distractions. Trained data collectors conducted one-on-one interviews, reading each item aloud and recording participants’ responses verbatim. Before data collection began, all data collectors completed standardized training that covered: (1) HIV-related clinical terminology; (2) principles of nonjudgmental communication; (3) methods for clarifying questions without leading the participant; (4) accurate transcription of responses; and (5) procedures for verifying medication lists and comorbidity histories. Mock interviews were conducted to ensure consistency between data collectors. To ensure reliability, all self-reported comorbidities and medications were verified against clinical records and pharmacy dispensing data. Inconsistencies were resolved through clarification with participants or clinicians. Completed questionnaires were reviewed for completeness, and data were double-entered using validation rules and logic checks. Research supervisors monitored data collection quality through periodic audits.

### 2.4. Data Analysis

Data were entered using EpiData 3.1 (EpiData Association, Odense, Denmark) and analyzed with SPSS 24 (IBM Corp., Armonk, NY, USA) and AMOS 24 (IBM Corp., Armonk, NY, USA) software [13]. Descriptive statistics were used to summarize participant characteristics. Normally distributed variables were analyzed using independent-samples t-tests or one-way ANOVA, while non-normally distributed variables were compared using the Mann–Whitney U test or Kruskal–Wallis test as appropriate. Pearson correlation analysis was conducted to examine the associations between continuous variables and medication adherence. Path analysis was conducted to examine the relationships among multimorbidity, polypharmacy, medication-related burden, PDDIs, and ART adherence. Model fit was evaluated using multiple goodness-of-fit indices, including the chi-square statistic and degrees of freedom ratio (χ^2^/df) and the standardized root mean square residual (SRMR). Following commonly accepted recommendations, acceptable model fit was indicated by χ^2^/df < 3.0 and SRMR < 0.08 for the low degrees of freedom and the use of observed variables only [14,15]. Statistical significance was set at a two-tailed *p*-value < 0.05. During data collection, strict quality control procedures were implemented. Questionnaires were reviewed on-site for completeness at the time of collection to ensure that no items were left unanswered. As a result, the final analytical dataset contained no missing questionnaire data.

## 3. Results

### 3.1. Participant Characteristics

A total of 301 older PLWH were included in the analysis. Participants were predominantly aged 50–59 years (62.5%), followed by those aged 60–69 years (24.9%) and 70–79 years (12.3%), with only one participant aged 80 years or older. Males accounted for 74.1% of the sample. Most participants resided in urban areas (63.5%) and were married (80.4%), while employment status and monthly income varied across the cohort. Overall, the ART adherence mean score was 14.82 ± 1.91. Among sociodemographic characteristics, gender was the only factor significantly associated with ART adherence, with female participants demonstrating higher adherence scores. Regarding HIV-related clinical characteristics, 91.7% (276/301) of participants had an undetectable viral load at their most recent assessment. The mean CD4 cell count at the time of HIV diagnosis was 238 ± 213 cells/μL, and the most recent mean CD4 cell count was 352 ± 209 cells/μL. More details were shown in Table 1.

### 3.2. Multimorbidity and Medication Use

The mean duration of ART was 54.61 ± 45.68 months. With respect to ART regimens, the vast majority of participants were receiving combinations that included nucleoside reverse transcriptase inhibitors (288/301, 95.7%). Regimens containing non-nucleoside reverse transcriptase inhibitors were used in 73.4% (221/301) of participants, while 17.9% (54/301) were prescribed regimens that included protease inhibitors. A small proportion (10/301, 3.3%) were receiving regimens containing integrase strand transfer inhibitors.

More than half of the participants (54.2%, 163/301) experienced multimorbidity in addition to HIV infection. Specifically, the three most common comorbidities were hypertension (*n* = 56), hyperlipidemia (*n* = 37), and diabetes mellitus (*n* = 28). Correspondingly, medications for cardiovascular and metabolic conditions were the most frequently prescribed non-HIV drugs and 159 participants were receiving cardiovascular system medications in addition to ART. Nearly one-third of the sample (29.2%, 88/301) were exposed to polypharmacy. The mean overall medication-related burden score was 105.55 ± 14.28, indicating a moderate to high level of perceived treatment burden among older PLWH. PDDIs were identified in 3.7% (11/301) of participants, including two contraindicated (red) interactions, one interaction requiring monitoring (amber), and eight weak (yellow) interactions.

### 3.3. Path Analysis

The proposed theory-driven path model demonstrated an acceptable fit to the data (χ^2^ = 6.998, df = 4, *p* = 0.136; χ^2^/df = 1.75). Given the low degrees of freedom and the use of observed variables only, model adequacy was primarily evaluated based on the standardized root mean square residual (SRMR = 0.0316), indicating acceptable residual fit. As shown in Table 2, multimorbidity was positively associated with polypharmacy (β = 0.233, *p* < 0.001) and with the presence of PDDIs (β = 0.284, *p* < 0.001). Multimorbidity was also directly associated with higher medication-related burden (β = 0.173, *p* = 0.004). Polypharmacy was significantly associated with PDDIs (β = 0.288, *p* < 0.001); however, its direct association with medication-related burden was not statistically significant (β = 0.023, *p* = 0.691). PDDIs were positively associated with medication-related burden (β = 0.193, *p* = 0.002), indicating that medication interaction risk was positively associated with perceived burden. With respect to ART adherence, medication-related burden was negatively associated with adherence scores (β = −0.151, *p* = 0.010). In contrast, neither polypharmacy (β = 0.005, *p* = 0.939) nor PDDIs (β = −0.082, *p* = 0.183) showed a significant direct association with ART adherence. Gender was included as a covariate because it was the only sociodemographic factor demonstrating a statistically significant association with antiretroviral therapy adherence in univariate analysis, and it was significantly associated with ART adherence (β = −0.145, *p* = 0.010).

**Figure 2:**
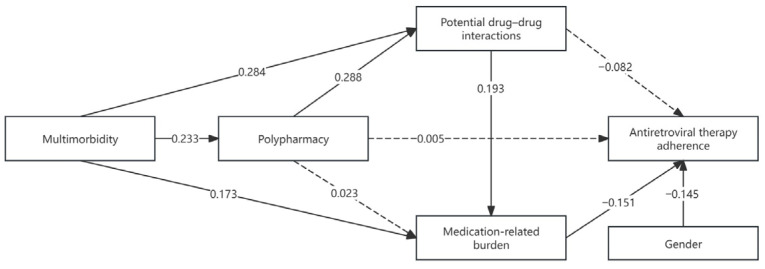
Path diagram of multimorbidity, polypharmacy, and medication-related factors on antiretroviral therapy adherence among older people living with HIV. Solid arrows represent significant paths (*p* < 0.05), dashed arrows represent non‑significant paths.

As shown in Figure 2, path analysis examined the associations among multimorbidity, medication use, medication-related burden, and ART adherence among older PLWH. Multimorbidity was positively associated with polypharmacy and with the presence of PDDIs. Both multimorbidity and PDDIs were significantly associated with higher medication-related burden, whereas polypharmacy showed no direct association with perceived burden. Medication-related burden was inversely associated with ART adherence, while neither polypharmacy nor PDDIs demonstrated a significant direct association with adherence.

## 4. Discussion

In this multicenter study of older PLWH, we found that multimorbidity and regimen complexity were associated with adherence primarily through their relationship with medication-related burden rather than through objective indicators alone. More than half of participants experienced multimorbidity, and nearly one-third met criteria for polypharmacy, reflecting the growing clinical complexity associated with aging with HIV. In an analysis from a Spanish cohort, polypharmacy prevalence exceeded 50% among individuals aged ≥50 years, with nervous system, cardiovascular, and metabolic medications being the most common concomitant therapies, underscoring the complex medication profiles encountered in such populations [16]. The relatively low prevalence of PDDIs in this study may reflect the predominance of regimens, limited use of protease inhibitors, and routine clinical medication review practices, which may have minimized clinically significant interactions. Some studies have reported higher rates of PDDIs in older HIV populations, up to 19% or more, particularly when polypharmacy is extensive and ART regimens include protease inhibitors or complex pharmacokinetic profiles [17]. However, PDDI prevalence varies widely depending on regimen composition and the interaction resources used. Our relatively low observed PDDI rate may therefore reflect common prescribing patterns within our setting as well as differences in database coverage, rather than indicating the absence of risk. The path analysis demonstrated that multimorbidity was associated with polypharmacy and PDDIs, which in turn contributed to greater medication-related burden. Importantly, medication-related burden emerged as the only factor directly associated with ART adherence, whereas polypharmacy and PDDIs showed no significant direct effects. These findings are consistent with the theoretical proposition that patients’ cognitive appraisal is associated with adherence behavior [18].

Our findings are consistent with prior research documenting high levels of multimorbidity and polypharmacy among older PLWH, driven by prolonged survival, chronic inflammation, and age-related comorbid conditions [4,17]. Previous studies examining the relationship between polypharmacy and ART adherence have reported mixed results, with some identifying negative associations and others finding no significant relationship [19,20,21]. The present study aligns with evidence suggesting that medication count alone may not adequately capture the challenges of long-term treatment management [22].

While PDDIs have been extensively studied from a pharmacological and safety perspective, fewer studies have examined their behavioral implications [23]. Our results show that PDDIs were associated with medication-related burden but not directly with ART adherence, highlighting the importance of patients’ perceptions of treatment complexity rather than objective interaction risk alone.

Medication-related burden has been increasingly recognized as a determinant of adherence in other chronic disease populations, yet it remains understudied in HIV care, particularly among older adults [24]. Previous studies in general geriatric populations have shown that higher numbers of daily medications are linked with greater risk of non-adherence and medication-related problems, suggesting that treatment complexity exerts a broader influence on adherence behaviors among older adults with multimorbidity [25]. Specifically, some studies report that although older PLWH may maintain high adherence to ART itself, adherence to concomitant non-ART medications tends to be lower as complexity increases [26]. By including medication-related burden as a conceptual intermediary construct, this study aligns with theoretical frameworks emphasizing cognitive appraisal processes in chronic illness management. Compared with prior HIV adherence studies focusing primarily on regimen characteristics, our findings underscore the added value of incorporating patient-reported measures to better capture the lived experience of long-term ART [27].

These findings have important implications for both clinical practice and public health strategies targeting aging HIV populations. The high prevalence of multimorbidity and polypharmacy underscores the need for integrated, multidisciplinary care models that address comorbid conditions alongside HIV management. Routine medication review and reconciliation should be prioritized to reduce unnecessary treatment complexity. The observed association of medication-related burden with adherence underscores of incorporating patient-reported assessments into routine HIV care. Objective indicators such as medication count or PDDIs may be insufficient to identify patients at risk of nonadherence. Interventions aimed at simplifying regimens, improving patient–provider communication, and addressing concerns related to side effects, daily life interference, and treatment overload may warrant further investigation in supporting long-term adherence. From a public health perspective, adherence support strategies for older PLWH should adopt a more patient-centered approach that acknowledges individual treatment experiences and cognitive appraisals. Integrating geriatric principles into HIV care and promoting interdisciplinary collaboration may help sustain optimal ART adherence and long-term viral suppression among aging populations [28].

Several limitations should be acknowledged. Although path analysis was used to estimate associations among observed variables with a continuous primary outcome, several predictors in the model were binary. Linear path modeling assumes linear relationships and homoscedastic residuals, and the inclusion of categorical predictors may not fully satisfy classical multivariate normality assumptions. While this approach remains appropriate for estimating conditional associations in cross-sectional data, the findings should be interpreted with methodological caution. Future studies based on a cohort study applying generalized structural equation modeling or alternative estimation techniques may further validate the robustness of the observed associations. The cross-sectional design precludes causal inference, and temporal relationships among key variables cannot be definitively established. Variables with very low prevalence, including PDDIs, may reduce effective statistical power for estimating their specific associations. The study was conducted in two HIV care settings within a single province in China, which may limit generalizability. Additionally, unmeasured factors such as cognitive function, mental health status, and social support may influence medication-related burden and adherence and warrant further investigation.

This study has several strengths. It focuses on an understudied but rapidly growing population of older PLWH and integrates objective clinical indicators with patient-reported medication-related burden within a theoretically informed analytical framework. The use of path analysis allowed for the simultaneous estimation of associations among multimorbidity, medication use, and adherence. Data quality was enhanced through interviewer-assisted data collection and verification of self-reported information against clinical records.

## 5. Conclusions

In conclusion, this study demonstrates that multimorbidity and polypharmacy are common among older PLWH and that their associations with ART adherence were primarily observed in relation to medication-related burden rather than objective treatment complexity alone. Medication-related burden represents an important correlate of adherence and should be routinely assessed in HIV care for aging populations. Interventions targeting patients’ experiences and perceptions of long-term treatment may be relevant for sustaining ART adherence and optimizing HIV outcomes in older adults.

## Figures and Tables

**Table 1:** Sociodemographic and clinical characteristics of older people living with HIV and comparisons of medication adherence across subgroups (*N* = 301).

Variables	*N* (%)/Mean ± SD	Medication Adherence (Mean ± SD)	*p* Value
Age (years)			
50–59	188 (62.5%)	14.81 ± 1.92	0.075
60–69	75 (24.9%)	14.51 ± 2.16	
70–79	37 (12.3%)	15.49 ± 1.04	
≥80	1 (0.3%)	16	
Gender			
Female	78 (25.9%)	15.19 ± 1.74	0.046
Male	223 (74.1%)	14.69 ± 1.96	
Residence			
Rural areas	110 (36.5%)	15.03 ± 1.79	0.145
Urban areas	191 (63.5%)	14.7 ± 1.98	
Marriage status			
Unmarried	8 (2.7%)	15.38 ± 1.06	0.183
Married	242 (80.4%)	14.74 ± 1.99	
Divorced	24 (8%)	14.67 ± 1.83	
Widowed	27 (9%)	15.52 ± 1.19	
Employment			
Temporary	54 (17.9%)	14.78 ± 1.68	0.526
Stable	72 (23.9%)	14.76 ± 1.83	
Farming	11 (3.7%)	15.82 ± 0.4	
Retired	61 (20.3%)	14.85 ± 1.82	
No job	103 (34.2%)	14.76 ± 2.21	
Monthly income (Chinese Yuan)			
≤1000	80 (26.6%)	14.68 ± 2.07	0.74
1001–3000	102 (33.9%)	14.76 ± 2.12	
3001–5000	75 (24.9%)	14.99 ± 1.48	
≥5001	44 (14.6%)	14.93 ± 1.8	
CD4 count at diagnosis (cells/μL)			
	238 ± 213	-	0.019
Most recent CD4 count (cells/μL)			
	352 ± 209	-	<0.001

For categorical variables, the *p*-values represent comparisons of medication adherence scores across subgroups using independent *t*-tests or one-way ANOVA, as appropriate. For continuous variables (CD4 count at diagnosis and most recent CD4 count), the *p*-values reflect the association between CD4 counts and medication adherence scores based on Pearson correlation analysis.

**Table 2:** Standardized path estimates from the structural equation model examining the effects of multimorbidity, polypharmacy, and medication-related factors on ART adherence among older people living with HIV.

Pathway			Standardized Estimate	SE	CR	*p*
Multimorbidity	→	Polypharmacy	0.233	0.09	4.148	<0.001
Multimorbidity	→	PDDIs	0.284	0.035	5.36	<0.001
Multimorbidity	→	Medication-related burden	0.173	0.88	2.92	0.004
Polypharmacy	→	PDDIs	0.288	0.022	5.428	<0.001
Polypharmacy	→	Medication-related burden	0.023	0.547	0.397	0.691
PDDIs	→	Medication-related burden	0.193	1.397	3.134	0.002
Polypharmacy	→	ART adherence	0.005	0.075	0.077	0.939
PDDIs	→	ART adherence	−0.082	0.189	−1.332	0.183
Medication-related burden	→	ART adherence	−0.151	0.008	−2.593	0.01
Gender	→	ART adherence	−0.145	0.246	−2.582	0.01

SE, standard error; CR, Critical Ratio; PDDIs, potential drug–drug interactions; ART, antiretroviral therapy. *p*-values are for statistical difference tests. Gender was included as a covariate because it was the only sociodemographic variable significantly associated with antiretroviral therapy adherence in univariate analyses. Paths were represented by →.

## Data Availability

The data presented in this study are available on request from the corresponding author due to privacy protection.

## Abbreviations

The following abbreviations are used in this manuscript:

ART	Antiretroviral Therapy
HIV	Human Immunodeficiency Virus
PDDIs PLWH	Potential Drug–Drug Interactions People living with HIV
CASE	Center for Adherence Support Evaluation
SRMR	Standardized Root Mean Square Residual

## References

[1] Longev L.H. (2022). Ageing with HIV. Lancet Healthy Longev..

[2] Smit M., Brinkman K., Geerlings S., Smit C., Thyagarajan K., Sighem A., de Wolf F., Hallett T.B. (2015). Future challenges for clinical care of an ageing population infected with HIV: A modelling study. Lancet Infect. Dis..

[3] Chauvin M., Sauce D. (2022). Mechanisms of immune aging in HIV. Clin. Sci..

[4] Yang C., Teh Y.E., Chua N.G.S., Lee K.L.S., Ng R.Q.M. (2024). An overview of multimorbidity and polypharmacy in older people living with HIV. Geriatr. Gerontol. Int..

[5] Bachmann P., Frahm N., Debus J.L., Mashhadiakbar P., Langhorst S.E., Streckenbach B., Baldt J., Heidler F., Hecker M., Zettl U.K. (2022). Prevalence and severity of potential drug–drug interactions in patients with multiple sclerosis with and without polypharmacy. Pharmaceutics.

[6] McCutcheon K., Nqebelele U., Murray L., Thomas T.S., Mpanya D., Tsabedze N. (2024). Cardiac and renal comorbidities in aging people living with HIV. Circ. Res..

[7] Sav A., King M.A., Whitty J.A., Kendall E., McMillan S.S., Kelly F., Hunter B., Wheeler A.J. (2015). Burden of treatment for chronic illness: A concept analysis and review of the literature. Health Expect..

[8] Hu X.J., Wang H.H., Li Y.T., Wu X.Y., Wang Y., Chen J.H., Wang J.J., Wong S.Y., Mercer S.W. (2022). Healthcare needs, experiences and treatment burden in primary care patients with multimorbidity: An evaluation of process of care from patients’ perspectives. Health Expect..

[9] de Cordova P.B., Reilly L.L., Pogorzelska-Maziarz M., Gerolamo A.M., Grafova I., Vasquez A., Johansen M.L. (2024). A theoretical framework for Acute Care Nurse Stress Appraisal: Application of the transactional model of stress and coping. J. Adv. Nurs..

[10] Lomax R.G. (2004). A Beginner’s Guide to Structural Equation Modeling.

[11] Katusiime B., Corlett S.A., Krska J. (2018). Development and validation of a revised instrument to measure burden of long-term medicines use: The Living with Medicines Questionnaire version 3. Patient Relat. Outcome Meas..

[12] Mannheimer S.B., Mukherjee R., Hirschhorn L.R., Dougherty J., Celano S.A., Ciccarone D., Graham K.K., Mantell J.E., Mundy L.M., Eldred L. (2006). The CASE adherence index: A novel method for measuring adherence to antiretroviral therapy. AIDS Care.

[13] Byrne B.M. (2001). Structural equation modeling with AMOS: Basic concepts. Applications, and Programming.

[14] Hu L.T., Bentler P.M. (1999). Cutoff criteria for fit indexes in covariance structure analysis: Conventional criteria versus new alternatives. Struct. Equ. Model. A Multidiscip. J..

[15] Kline R.B. (2023). Principles and Practice of Structural Equation Modeling.

[16] López López A., Pérez González A., Alonso Domínguez J., Ocampo A., Miralles C., Morano L., Aguayo Arjona J., Martínez López de Castro N., Poveda E. (2026). High prevalence of polypharmacy and nervous system medications in people with HIV: A cross-sectional analysis. Sci. Rep..

[17] Demirbas N.D., Diktas H., Gul O., Derin O., Ozgur U., Aksu S.B., Atasoy Tahtasakal C., Oncul A., Yildiz Sevgi D., Dokmetas I. (2025). Aging with HIV: Multimorbidity and polypharmacy burden. AIDS Care.

[18] Poręba-Chabros A., Kolańska-Stronka M., Mamcarz P., Mamcarz I. (2022). Cognitive appraisal of the disease and stress level in lung cancer patients. The mediating role of coping styles. Support. Care Cancer.

[19] Zheng C., Meng J., Xiao X., Xie Y., Zhao D., Wang H. (2022). Polypharmacy, Medication-Related Burden and Antiretroviral Therapy Adherence in People Living with HIV Aged 50 and Above: A Cross-Sectional Study in Hunan, China. Patient Prefer. Adherence.

[20] Bevilacqua K., Brinkley C., McGowan J., Wallach F., Schwartz R. (2022). “We are Getting Those Old People Things.” Polypharmacy Management and Medication Adherence Among Adult HIV Patients with Multiple Comorbidities: A Qualitative Study. Patient Prefer. Adherence.

[21] Elbur A., Ghebremichael M., Konkle-Parker D., Jones D., Collins S., Adimora A., Schneider M., Cohen M., Tamraz B., Plankey M. (2023). Dual Trajectories of Antiretroviral Therapy Adherence and Polypharmacy in Women with HIV in the United States. AIDS Res. Ther..

[22] Sukumaran L., Winston A., Marzolini C., Khoo S., Boffito M., Naous N., Sabin C.A. (2025). Polypharmacy in HIV: Rethinking what counts and why it matters. HIV Med..

[23] Marques L., Vale N. (2025). A Review on New Frontiers in Drug-Drug Interaction Predictions and Safety Evaluations with In Vitro Cellular Models. Pharmaceutics.

[24] Selvakumar D., Sivanandy P., Ingle P.V., Theivasigamani K. (2023). Relationship between treatment burden, health literacy, and medication adherence in older adults coping with multiple chronic conditions. Medicina.

[25] Huang J., Wang H.H.X., Zheng Z., Wong M.C.S. (2020). Medication adherence among the older adults: Challenges and recommendations. Hong Kong Med. J..

[26] Sarma P., Cassidy R., Corlett S., Katusiime B. (2023). Ageing with HIV: Medicine Optimisation Challenges and Support Needs for Older People Living with HIV: A Systematic Review. Drugs Aging.

[27] Cohen J., Beaubrun A., Bashyal R., Huang A., Li J., Baser O. (2020). Real-world adherence and persistence for newly-prescribed HIV treatment: Single versus multiple tablet regimen comparison among US medicaid beneficiaries. AIDS Res. Ther..

[28] Kokorelias K.M., Grosse A., Zhabokritsky A., Sirisegaram L. (2023). Understanding geriatric models of care for older adults living with HIV: A scoping review and qualitative analysis. BMC Geriatr..

